# Gene Signature and Identification of Clinical Trait-Related m^6^ A Regulators in Pancreatic Cancer

**DOI:** 10.3389/fgene.2020.00522

**Published:** 2020-07-10

**Authors:** Jie Hou, Zhan Wang, Hong Li, Hongzhi Zhang, Lan Luo

**Affiliations:** The People’s Hospital of Baoan Shenzhen, The 8th people’s Hospital of Shenzhen, The Affiliated Baoan Hospital of Southern Medical University, Shenzhen, China

**Keywords:** m^6^A regulators, pancreatic cancer, prognostic model, biomarker, clinical traits

## Abstract

Pancreatic cancer (PC) has a very poor prognosis and is usually diagnosed only at an advanced stage. The discovery of new biomarkers for PC will help in early diagnosis and a better prognosis for patients. Recently, N6-methyladenosine (m^6^A) RNA modifications and their regulators have been implicated in the development of many cancers. To investigate the functions and mechanisms of m^6^A modifications in the development of PC, 19 m^6^A regulators, including m^6^A-methyltransferases (ZC3H13, RBM15/15B, WTAP, KIAA1429, and METTL3/14), demethylases (FTO and ALKBH5), and binding proteins (YTHDF1/2/3, YTHDC1/2, IGF2BP1/2/3, HNRNPC, and HNRNPA2B1) were analyzed in 178 PC tissues from the cancer genome atlas (TCGA) database. The results were verified in PC cell lines Mia-PaCa-2, BXPC-3, and the control cell line HDE-CT. The m^6^A regulators-based sample clusters were significantly related to overall survival (OS). Further, lasso regression identified a six-m^6^A-regulator-signature prognostic model (KIAA1429, HNRNPC, METTL3, YTHDF1, IGF2BP2, and IGF2BP3). Model-based high-risk and low-risk groups were significantly correlated with OS and clinical traits (pathologic M, N, and clinical stages and vital status). The risk signature was verified as an independent prognostic marker for patients with PC. Finally, gene set enrichment analysis revealed m^6^A regulators (KIAA1429, HNRNPC, and IGF2BP2) were related to multiple biological behaviors in PC, including adipocytokine signaling, the well vs. poorly differentiated tumor pathway, tumor metastasis pathway, epithelial mesenchymal transition pathway, gemcitabine resistance pathway, and stemness pathway. In summary, the m6A regulatory factors which related to clinical characteristics can be involved in the malignant progression of PC, and the constructed risk markers may be a promising prognostic biomarker that can guide the individualized treatment of PC patients.

## Introduction

Pancreatic cancer (PC) is one of the most lethal malignant neoplasms and has become one of the leading causes of cancer-related deaths in developed countries (Ilic and Ilic, 2016). About 85% of PCs are adenocarcinoma, and less than 5% are pancreatic endocrine tumors (Wolfgang et al., 2013). There are usually no noticeable symptoms in the early stages of PC. When symptoms are specific enough to suggest PC, the disease might have reached an advanced stage. By the time of diagnosis, PC has often spread or metastasized to other parts of the body (Mohammed et al., 2014). With the development of medical techniques, PC can be diagnosed by ultrasound or computed tomography combined with blood tests and examination of tissue samples (biopsies). However, screening the general population for the early stage of the disease is not effective (Welinsky and Lucas, 2017). Pancreatic cancer can be treated with surgery, chemotherapy, targeted therapy, radiotherapy, palliative care, immunotherapy, or a combination of these based on the cancer stage (Mcguigan et al., 2018). With the current treatment methods pancreatic adenocarcinoma has a very poor prognosis: only 25% of patients with PC survive one year and 5 year-overall survival (OS) is lower than 5% (Ansari et al., 2015). Current treatment and diagnostic methods are not enough for the management of PC. Therefore, key goals for PC research are to develop novel prognostic markers, improve the early diagnostic rate, and find new targets for molecular targeted therapy. N6-methyladenosine (m^6^A), a potential biomarker, is a chemical modification present in multiple RNA species, which take part in various biological processes in cancer (Liu et al., 2018).

N6-methyladenosine regulators are involved in more than 60% of all RNA [messenger (mRNA), transport RNA (tRNA), and ribosomal RNA (rRNA)] modifications, which is an intense area of research for post-transcriptional regulation including translation, mRNA splicing, and mRNA stability (Yu et al., 2018). The level of modification of transcripts with m^6^A is regulated by methyltransferases, binding proteins and demethylases (Koh et al., 2019). The methyltransferases (including ZC3H13, RBM15, RBM15B, KIAA1429, METTL3/14, and WTAP), act as “writers,” and add the methyl group to the nitrogen on the sixth carbon of the aromatic ring of an adenosine residue (Meyer and Jaffrey, 2017). The cellular m^6^A status is reverted by demethylases (FTO, and ALKBH5; called “erasers”), and is recognized by m^6^A-binding proteins (HNRNPC, YTHDF1/2/3, YTHDC1/2, IGF2BP1/2/3, and HNRNPA2B1; called “readers”) (Zaccara et al., 2019). N6-methyladenosine, a potential biomarker, is a chemical modification present in multiple RNA species, which take part in various biological processes in cancer (Liu et al., 2018). The dysregulation of m^6^A regulators is involved in the occurrence and development of multiple cancers, including bladder cancer, prostate cancer, head and neck squamous cell carcinoma, gastric cancer, breast cancer, hepatocellular carcinoma, and colorectal cancer (Hong, 2018). For example, METTL14 which suppresses colorectal cancer progression via regulating m^6^A-dependent miR-375/yes-associated protein 1 (YAP1) pathway, is downregulated in colorectal cancer tissues and cell lines (Chen et al., 2020). FTO, a key m^6^A demethylase, is up-regulated in human breast cancer and is significantly associated with poor survival rates (Niu et al., 2019). FTO mediates m^6^A demethylation in the 3’UTR of BNIP3 mRNA and induces its degradation via an YTHDF2 independent mechanism, which indicates that FTO can serve as a novel potential therapeutic target for breast cancer (Niu et al., 2019). It has also been reported that IGF2BP2 regulates lncRNA DANCR through m6A modification, and IGF2BP2 and DANCR jointly promote the stemness-like characteristics of cancer and the pathogenesis of PC (Hu et al., 2019). Although more and more studies have shown that m6A regulatory factors play a crucial role in the pathogenesis and development of cancer, the fundamental relationship between m6A regulatory factors and PC remains unclear (Xia et al., 2019). The construction of prognostic signal based on m6A regulators that predicting the prognosis of PC will be helpful for prediction, prevention and personalized treatment.

This study used ConsensusClusterPlus to find that m^6^A regulators were closely related to PC OS rates in different clusters. Furthermore, lasso regression was used to identify a six-gene signature model (KIAA1429, HNRNPC, METTL3, YTHDF1, IGF2BP2, and IGF2BP3). Most of the genes identified were consistent with previous data (Taketo et al., 2018). For example, the m^6^A eraser ALKBH5, which was indicated as a potential therapeutic target for PC, was downregulated in PC cells and immortalized human pancreatic duct epithelial (HPDE6-C7) cells (He et al., 2018). Immunohistochemistry (IHC), western blots, and RT-qPCR were used to detect the expression of METTL3 in PC, and the results showed that METTL3 protein and mRNA levels were significantly higher in tumor samples than in paracancer samples. Down-regulation of METTL3 reduced the proliferation, invasion and migration of PC cell lines (Xia et al., 2019). While it is known that m^6^A plays important roles in different types of cancers, the available clinical trait-related m^6^A regulator studies in PC are insufficient. Single-gene analysis are used to predict prognosis and to guide therapy in cancer. However, RNA-Seq is helpful for the construction of a prediction model using multiple genes. Here, we analyzed the gene signatures in different PC cell lines and identified clinical trait-related m^6^A regulators in PC. Additionally, potential related enrichment pathways of m^6^A regulators might be useful to further study their mechanisms of action.

## Materials and Methods

### Data Sources

RNA-seq transcriptome data, the corresponding clinical data, and large-scale cancer patient information for 178 patients with PC were obtained from the cancer genome atlas (TCGA) database^1^. The m^6^A regulator genes include ZC3H13, RBM15/15B, KIAA1429, METTL14, YTHDC1/2, WTAP, METTL3, FTO, ALKBH5, YTHDF1/2/3, HNRNPA2B1, IGF2BP1/2/3, and HNRNPC. The corresponding clinical data include age at initial pathologic diagnosis (patients were aged 35–88), documented alcohol history (yes or no), alcoholic exposure category (daily drinker, weekly drinker, occasional drinker, social drinker, and non-drinker), anatomic neoplasm subdivision (body of pancreas, head of pancreas, tail of pancreas, and other parts), family history of cancer (yes or no), gender (male and female), history of chronic pancreatitis (yes or no), history of diabetes (yes or no), count of lymph nodes examined (from 1 to 57), neoplasm histologic grade (G1, G2, and G3), pathologic M (M represents tumor metastasis, including M0, M1,and MX), pathologic N (N represents tumor lymph node metastasis, including N0, N1, N2, and NX), pathologic T (T represents tumor size, including T1, T2, T3,T4, and TX), pathologic stage (Stages I, II, III, and IV), vital status (alive or dead).

We systematically searched for PC gene expression datasets that were publicly available and reported full clinical annotations. we download data from GSE28735 “Microarray gene-expression profiles of 45 matching pairs of pancreatic tumor and adjacent non-tumor tissues from 45 patients with pancreatic ductal adenocarcinoma” to validate the reliability of the built model. The raw data from the microarray datasets generated by Affymetrix and Illumina were downloaded from the Gene Expression Omnibus^2^.

### Protein–Protein Interactions Network Construction and Correlation Analysis

The STRING database^3^ was used for analyzing the protein–protein interactions (PPI) among m^6^A regulators. The association among different m^6^A regulators was revealed by Spearman correlation coefficient with R package.

### Cell Lines and Cell Culture

Two PC cell lines (Mia-PaCa-2 and BXPC-3) and one control cell line (HDE-CT) were purchased from China Center for Type Culture Collection (CCTCC, Shanghai, China). HDE-CT is a normal human pancreatic cell line and is cultured in DMEM medium (Corning, NY, United States) supplemented with 10% fetal bovine serum (GIBCO, South America, NY, United States). Mia-PaCa-2 with a KRAS mutation and BXPC-3 with wild type KRAS are human PC cell lines. Mia-PaCa-2 was cultured in DMEM medium with 10% fetal bovine serum, and BXPC-3 was cultured in RPMI-1640 medium with 10% fetal bovine serum. All cell lines were maintained in 5% CO_2_ atmosphere at 37°C.

### RNA Extraction and qRT-PCR Verification

Total RNA of the four PC cell lines (Mia-PaCa-2 and BXPC-3) and HDE-CT were extracted with an RNA extraction kit (QIAGEN) according to the manufacturer’s instructions. Briefly, 1 × 10^7^ cells were collected and lysed for 10 min, genomic DNA was removed with an adsorption column, the samples were washed once with 75% ethyl alcohol and twice with wash buffer, and the samples were resuspended in RNA-grade enzyme-free water. Total RNA was reversely transcribed into cDNA and used to perform quantitative real-time PCR (qRT-PCR) with SYBR Premix ExTaq (TaKaRa). GAPDH was used as a reference gene. Primers (Table 1) were synthesized by Sangon Biotech (Shanghai, China).

**TABLE 1:** The list of RNA molecules that were assessed on the cell lines (note:F forward, R reverse).

**Primer name**	**Primer sequence (from 5′ to 3′)**
ZC3H13-F	GATCAGTTAAAGCGTGGAGAAC
ZC3H13-R	CTCTCTGTCGTGTTCATATCGA
FTO-F	GTTCACAACCTCGGTTTAGTTC
FTO-R	CATCATCATTGTCCACATCGTC
ALKBH5-F	GCAAGGTGAAGAGCGGCATCC
ALKBH5-R	GTCCACCGTGTGCTCGTTGTAC
KIAA1429-F	GCAACTTCAGGCATTAAGTTCA
KIAA1429-R	GTATTGCCTTGTCGAATCTGTC
METTL14-F	CAGGCTGGCTCACAGTTGGAC
METTL14-R	TTCCACCTCTTCCTCCACCTCTG
METTL3-F	CTTCAGCAGTTCCTGAATTAGC
METTL3-R	ATGTTAAGGCCAGATCAGAGAG
RBM15-F	GGCTGCCTGAGGAGAGTGGAG
RBM15-R	CGGCTACTGCTCAATTCTGGACTG
RBM15B-F	ATCTTTCAGAGTACGCTCAGAC
RBM15B-R	CTAGGATATGCATAGACGTGGG
WTAP-F	CTGACAAACGGACCAAGTAATG
WTAP-R	AAAGTCATCTTCGGTTGTGTTG
YTHDC1-F	AGTGACTCTGGTTCTGAATCTG
YTHDC1-R	CTGGTTTGATCTTTTCGGACAG
YTHDC2-F	GAGAATTGGGCTGTCGTTAAAG
YTHDC2-R	TGAAGCAGGATGAAATCGTACT
YTHDF2-F	ACTTCTCAGCATGGGGAAATAA
YTHDF2-R	TATTCATGCCAGGAGCCTTATT
YTHDF3-F	TCAACCACCACAACCACAGCAG
YTHDF3-R	TGAAGCACTGACAGGTACAACACC
IGF2BP1-R	GGGGTGGAATATTTCGGATTTG
IGF2BP1-F	GATGAAGGCCATCGAAACTTTC
IGF2BP2-F	GATGAACAAGCTTTACATCGGG
IGF2BP2-R	GATTTTCCCATGCAATTCCACT
IGF2BP3-F	GAGGCGCTTTCAGGTAAAATAG
IGF2BP3-R	AATGAGGCGGGATATTTCGTAT
YTHDF1-F	ATGACAATGACTTTGAGCCCTA
YTHDF1-R	AGGGAGTAAGGAAATCCAATGG
HNRNPA2B1-F	GCTTAAGCTTTGAAACCACAGA
HNRNPA2B1-F	GCTTAAGCTTTGAAACCACAGA
HNRNPC-F	ACAGATCCTCGCTCCATGAACTCC
HNRNPC-R	TTCTGCCATCCTCTCCTGCTACAG
GAPDH-F	CTGCACCACCAACTGCTT
GAPDH-R	TTCTGGGTGGCAGTGATG

### Consensus Clustering for PC Tissues

Pancreatic cancer tissues with expression information for m^6^A regulator genes (ZC3H13, RBM15, RBM15B, KIAA1429, YTHDC1, YTHDC2, METTL3, METTL14, WTAP, FTO, ALKBH5, YTHDF1, YTHDF2, YTHDF3, IGF2BP1, IGF2BP2, IGF2BP3, HNRNPA2B1, and HNRNPC) were clustered with a hierarchical agglomerative consensus. Clustering was based on Ward’s linkage and Euclidean distance methods. Unsupervised clustering methods use the proportion of ambiguous clustering (PAC) to infer optimal K (K-means) in order to identify and classify patients for further analysis (Lock and Dunson, 2013). Cluster analysis was performed using the ConsensusClusterPlus R package with cycle computation for 1000 times to ensure the stability and reliability of the classification (Wilkerson and Hayes, 2010). The Kaplan–Meier method was used for the OS analysis in different clusters.

### Lasso Regression for PC Tissues

Lasso is a regression analysis method that performs both variable selection and regularization in order to enhance the prediction accuracy and interpretability of the statistical model it produces. The best subset selection and the connections between lasso coefficient estimates can be identified to construct a prognostic model (Alhamzawi and Ali, 2018). Lasso regression was constructed to examine the relationship between gene signatures and PC risk. Further, clinical characteristics associated with OS were analyzed in patients with PC using Cox regression (including univariate and multivariate models) and the Kaplan–Meier method to evaluate the availability of the prognostic model. Pheatmap R package was used to correlate clinical data with the risk score (high or low).

### Gene Set Enrichment Analysis for KIAA1429, HNRNPC, and IGF2BP2 in PC Tissues

Gene set enrichment analysis (GSEA) is widely used to analyze genome or proteome data, linking disease phenotypes with many different functional gene sets. The 178 patients with PC were divided into high expression groups and low expression groups according to the median expression values of KIAA1429, HNRNPC, and IGF2BP2. Two groups of TCGA data were analyzed by GSEA. Gene set enrichment analysis was also conducted in different sample risk groups based on the LASSO regression model. The 178 patients with PC were divided into high risk score group and low risk score group according to the median value of risk score.

### Transient Transfection and Cell Proliferation Assay

The cells Mia-PaCa-2 and BXPC-3 were seeded in 6-well plates at 30–50% density. Transient transfection was performed with Lipo-fectamine 3000 reagents according to the manufacturer’s instructions (Invitrogen, United States). For all the experiments, cells were collected at 24–48 h after transfection. After transfection, the cells were seeded in 96-well plates and cultured for 1–3 days according to 5000/well. On the indicated days, the CCK8 reagent (Sigma, St. Louis, MO, United States) was added, and the cells were incubated for 2 h at 37°C. The absorbance at 450 nm for each sample was measured using a microplate reader of Bio-Tek ELx800 (United States). For the colony formation assay, After transfection for 48 h, cells were used to measure DNA synthesis with a Cell-LightTM EdU imaging detecting kit (RiboBio, Guangzhou, China) according to the manufacturer’s instructions.

### Statistical Analysis

Gene expression data of FPKM form is used as input. WilcoxTest is used to get the *p* value for different expression between different clusters. The relationships between clusters or different risk score groups were analyzed using the Chi-square test. In all cases, *p* < 0.05 was considered statistically significant. Spearman correlation coefficient was calculated for the molecular pairing between m^6^A regulator genes. The student’s *t*-test in SPSS 13.0 (SPSS Inc., Chicago, United States) was used to assess the expression differences between HDE-CT and PC cancer cells. Each experiment was repeated at least three times. Benjamini-Hochberg for multiple testing, and false discovery rate (FDR) were calculated to correct the *p*-value in GSEA.

## Results

### Consensus Clustering for PC Tissues Based on the Expression of m6A Regulators

**FIGURE 1:**
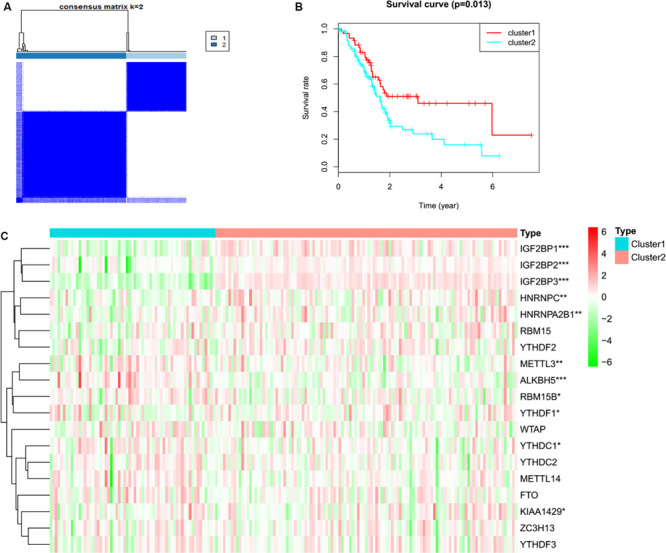
Consensus clustering and heatmap. **(A)** Consensus clustering for PC tissues based on the expression of m^6^A regulators (*k* = 2). **(B)** The overall survival in cluster 1 was significantly shorter than that in cluster 2. **(C)** Expression differences of m^6^A RNA methylation regulators in pancreatic cancer based on TCGA data. Red and green represent relatively high or low expression, respectively. *p < 0.05, **p < 0.01, and ***p < 0.001.

To determine whether the expression levels of m^6^A regulators were associated with PC prognosis, the TCGA PC cohort was clustered into different groups by consensus expression of m^6^A regulators with the ConsensusClusterPlus R package. Gene signatures of m^6^A regulators in PC are shown in Supplementary Table S1. When the consensus matrix *k* value was equal to 2, there was no crossover between PC samples (Figure 1A, Supplementary Figure S1 and Supplementary Table S2). The OS difference between different clusters was calculated by the Kaplan–Meier method and log-rank test (Figure 1B and Supplementary Table S2). A heatmap was generated to visualize the expression pattern of m^6^A regulators between different clusters (Figure 1C). The expression levels of RBM15B (*p* = 0.037), HNRNPC (*p* = 0.001), METTL14 (*p* = 0.007), METTL3 (*p* = 0.005), YTHDC1 (*p* = 0.049), KIAA1429 (*p* = 0.010), ALKBH5 (*p* = 3.50E-06), YTHF2 (*p* = 0.038), HNRN *p* A2B1 (*p* = 0.003), IGF2BP1 (*p* = 1.22E-11), IGF2BP2 (*p* = 1.10E-05), and IGF2BP3 (*p* = 2.34E-27) showed a significant dysregulation in tumor samples between different clusters.

### The Interaction and Correlation Among the m^6^A Regulators

**FIGURE 2:**
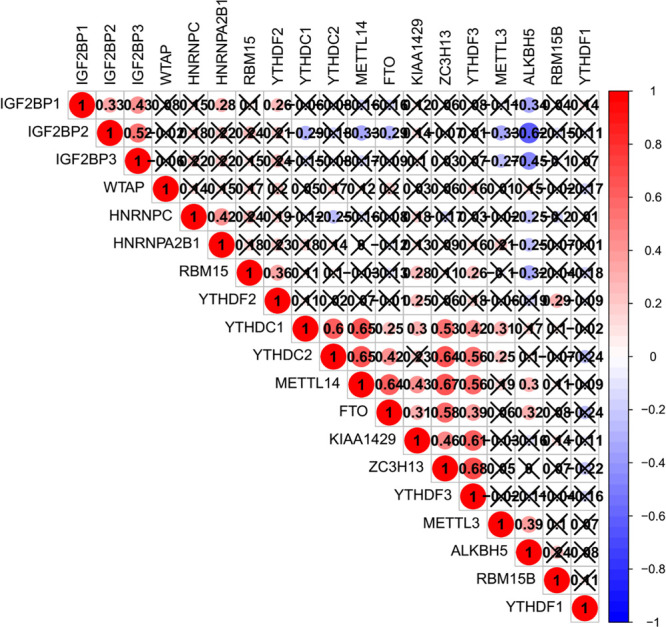
Co-expression of m^6^A regulator genes.

**FIGURE 3:**
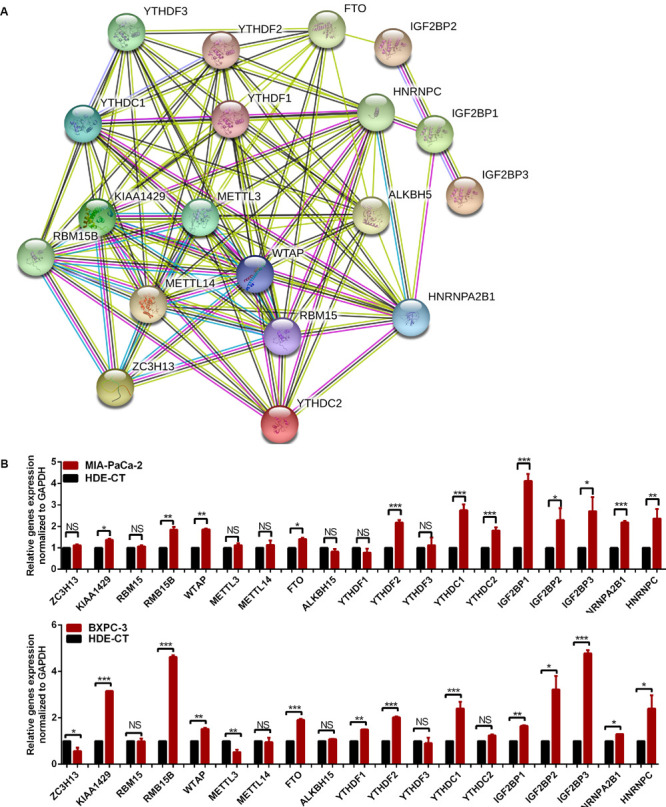
The relationship of m^6^A regulators in pancreatic cancer (PC) tissues. **(A)** Protein–protein interaction network of m^6^A regulator proteins. **(B)** Gene signature of m^6^A regulators in PC cell lines. *p < 0.05, **p < 0.01, and ***p < 0.001.

The relationship between m^6^A regulators were further supported by the correlation analysis. Some highly correlated (|correlation coefficient| ≥ 0.5, *p* < 0.05) m^6^A regulator pairs were identified, including IGF2BP2 and IGF2BP3, IGF2BP2 and ALKBH5, YTHDC1 and YTHDC2, YTHDC1 and METTL14, YTHDC1 and ZC3H13, YTHDC2 and METTL14, YTHDC2 and ZC3H13, YTHDC2 and YTHDF3, METTL14 and FTO, METTL1 and ZC3H13, METTL14 and YTHDF3, FTO and ZC3H13 (Figure 2 and Supplementary Table S3). The interactions among the 19 m^6^A regulators are shown in Figure 3A. All m^6^A regulators have interactions in the same network. The results of the interaction network showed that IGF2BP1 and IGF2BP3, WTAP and KIAA1429, HNRNPC and HNRNPA2B1, WTAP and ZC3H13, METTL14 and METTL3, KIAA1429 and ZC3H13, METTL14 and WTAP, WTAP and METTL3, METTL14 and KIAA1429, METTL3 and KIAA1429 have high combined score (>0.99).

### Gene Signature of m^6^A Regulators in PC Cell Lines

The expression of m^6^A regulators, including the m^6^A methyltransferases, the demethylases, and the m^6^A-binding proteins were analyzed by qRT-PCR in the PC cell lines, Mia-PaCa-2 and BXPC-3, and the control cell line HDE-CT. The results showed that some m^6^A regulators were differentially expressed in PC and control cell lines (Figure 3B).

### Lasso Regression Identified the Six-Gene Signature Prognostic Model

**FIGURE 4:**
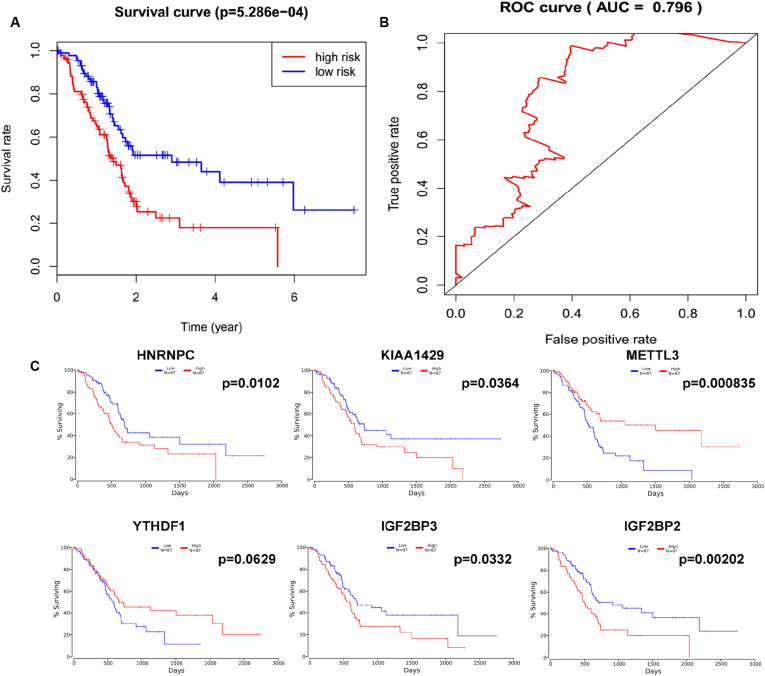
Lasso regression identified a six-gene signature prognostic model. **(A)** Overall survival analysis of the high risk score and low risk score groups. **(B)** ROC curve was used to evaluate the prediction efficiency of the prognostic signature. **(C)** Kaplan–Meier (KM) survival curve of KIAA1429, HNRNPC, METTL3, YTHDF1, IGF2BP2, and IGF2BP3 in pancreatic cancer.

**FIGURE 5:**
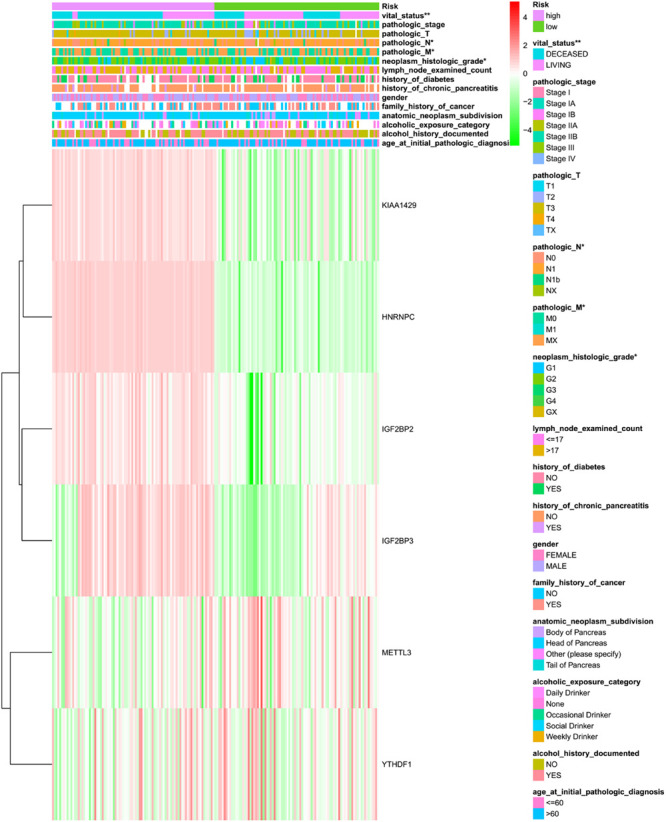
The heatmap of sample risk groups and related pancreatic cancer clinical characteristics. Age at initial pathologic diagnosis (patients were aged 35–88), alcohol history documented (yes or no), alcoholic exposure category (daily drinker, weekly drinker, occasional drinker, social drinker, and non-drinker), anatomic neoplasm subdivision (body of pancreas, head of pancreas, tail of pancreas, and other parts), family history of cancer (yes or no), gender (male and female), history of chronic pancreatitis (yes or no), history of diabetes (yes or no), count of lymph nodes examined (from 1 to 57), neoplasm histologic grade (G1, G2, and G3), pathologic M (M represents tumor metastasis, including M0, M1,and MX), pathologic N (N represents tumor lymph node metastasis, including N0, N1, N2, and NX), pathologic T (T represents tumor size, including T1, T2, T3,T4, and TX), pathologic stage (Stages I, II, III, and IV), vital status (alive or dead). *p < 0.05 and **p < 0.01.

In order to determine the optimal prognostic model, lasso regression was performed using the glmnet R package. Lasso regression is a generalized linear model, and the adjustment degree of lasso regression complexity is controlled by lambda. The optimal six-gene signature prognostic model was identified when log (lambda) was between −2 and −3 (Supplementary Figures S2A,B), where the coefficient of KIAA1429 was 0.28, the coefficient of HNRNPC was 0.34, the coefficient of METTL3 was −0.11, the coefficient of YTHDF1 was −0.37, the coefficient of IGF2BP2 was 0.28, and the coefficient of IGF2BP3 was 0.04. According to the median risk score, patients were divided into low- and high-risk groups (Supplementary Table S4). There was a significant difference in the OS rate between the two groups, and the OS rate of the high-risk group was significantly lower than that of the low-risk group (Figure 4A, *p* = 5.286e-04). A Receiver Operating Characteristiccurve (ROC) was used to evaluate the prediction efficiency of the prognostic signature. The prognostic signature model showed good prediction efficiency with the value of the area under the ROC curve (AUC) equal to 0.796 (Figure 4B). Additionally, the KM plotter showed that the six selected m^6^A regulators were significantly related with OS according to OncoLnc^4^ (Figure 4C). Importantly, the heat map shows the expression of the six selected m^6^A regulators and clinicopathological variables in the high- and low-risk groups. Significant differences were found for the neoplasm histologic grade, pathologic M stage, pathologic N stage, pathologic stage, and vital status between high- and low-risk groups (Figure 5 and Supplementary Table S5).

### The Effect of m^6^A Regulators on PC Prognosis

**FIGURE 6:**
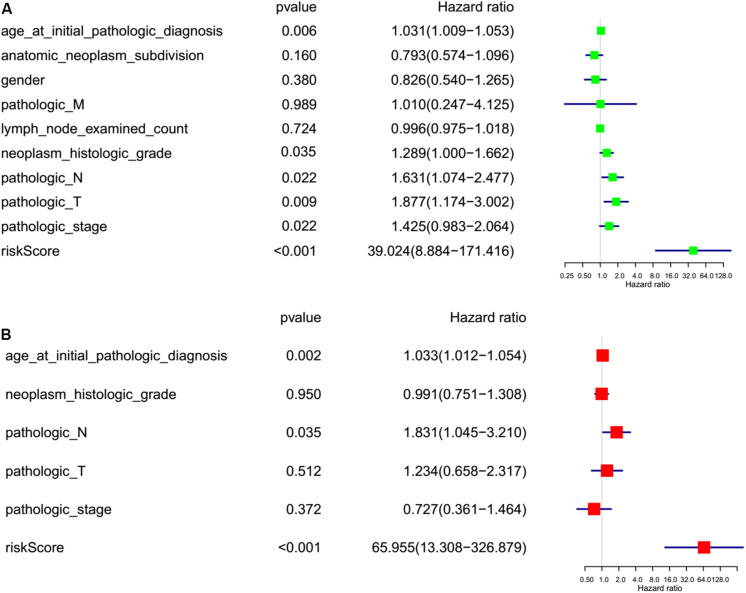
Risk factor analyses for pancreatic cancer (PC). **(A)** Univariate analysis of risk factors for PC. **(B)** Multivariate analysis of risk factors for PC. Age at initial pathologic diagnosis (>60 vs. <60), anatomic neoplasm subdivision (body of pancreas, head of pancreas, tail of pancreas, and other parts), gender (male vs. female), count of lymph nodes examined (>17 vs. <17), neoplasm histologic grade (G1, G2, and G3), pathologic M (M represents tumor metastasis, including M0, M1,and MX), pathologic N (N represents tumor lymph node metastasis, including N0, N1, N2, and NX), pathologic T (T represents tumor size, including T1, T2, T3,T4, and TX), pathologic stage (Stages I, II, III, and IV), risk score (high risk score group vs. low risk score group).

To investigate the effect of m^6^A regulators on PC prognosis, we performed Cox univariate (Figure 6A) and multivariate analysis (Figure 6B). The six-gene signature was consistent with the single-factor analysis of genes using Cox regression. The univariate analysis revealed that age at initial pathologic diagnosis [hazard ratio (HR): 1.031; 95% confidence interval (CI): 1.009–1.053 *p* = 0.006], neoplasm histologic grade [hazard ratio (HR): 1.289; 95% confidence interval (CI): 1.000–1.662; *p* = 0.035], pathologic N stage [hazard ratio (HR): 631; 95% confidence interval (CI): 1.074–2.477; *p* = 0.022], pathologic T stage [hazard ratio (HR): 1.877; 95% confidence interval (CI): 1.174–3.002; *p* = 0.009] pathologic stage [hazard ratio (HR): 1.425; 95% confidence interval (CI): 0.983–2.064; *p* = 0.022], and risk score [hazard ratio (HR): 30.024; 95% confidence interval (CI): 8.884–171.416; *p* < 0.001] were correlated significantly with a poor OS (Figure 6A). The multivariate analysis revealed that age at initial pathologic diagnosis [hazard ratio (HR): 1.033; 95% confidence interval (CI): 1.012–1.054; *p* = 0.002], pathologic N stage [hazard ratio (HR): 1.831; 95% confidence interval (CI): 1.045–3.210; *p* = 0.035], and risk score [hazard ratio (HR): 65.955; 95% confidence interval (CI): 13.308–326.879; *p* < 0.001] were correlated significantly with a poor OS (Figure 6B). The factor of risk score based on the optimal six-gene signature prognostic model was significant both at univariate and multivariate analyses.

### GSEA Analysis Provided Insight Into Pathways of m^6^A Regulators

**FIGURE 7:**
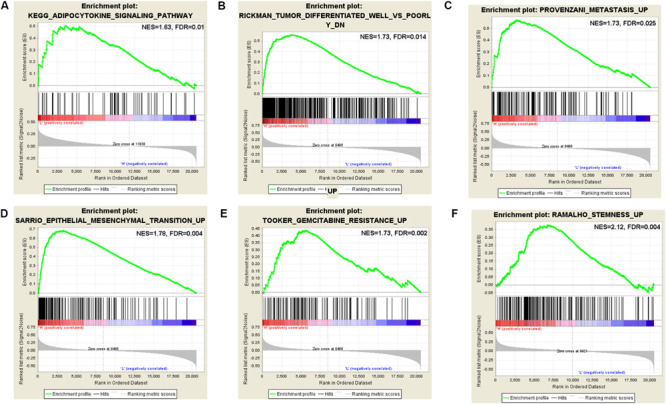
Gene set enrichment analysis (GSEA) for KIAA1429, HNRNPC, and IGF2BP2. **(A)** GSEA enriched the adipocytokine signaling pathway of HNRNPC. **(B)** GSEA enriched the well vs. poorly differentiated tumor pathway of IGF2BP2. **(C)** GSEA enriched the tumor metastasis pathway of IGF2BP2. **(D)** GSEA enriched the epithelial mesenchymal transition pathway of IGF2BP2. **(E)** GSEA enriched the gemcitabine resistance pathway of IGF2BP2. **(F)** GSEA enriched the stemness pathway of KIAA1429.

According to the coefficient of m^6^A regulators in the six-gene signature prognostic model and OS analysis, the GSEA result of HNRNPC showed that it is significantly related to phospholipase-c mediated cascade, type II diabetes mellitus, signaling by FGFR, downstream signaling of activated FGER, calcium signaling pathway, signaling by FGFR in disease, adipocytokine signaling pathway, vascular smooth muscle contraction, and metastasis. The GSEA result of IGF2BP2 showed that it is significantly related to metastasis, CREBBP targets, docetaxel resistance, hypoxia, BRCA1 targets, base excision repair, TAP63 pathway, etoposide sensitivity, epithelial mesenchymal transition, gemcitabine resistance, cisplatin resistance, gefitinib resistance, tumor differentiated well vs. poorly, and SFRP2 targets. The GSEA result of KIAA1429 showed that it is significantly related to CD5 targets, stemness, ubiquitin mediated proteolysis, YY1 targets, UV response via ERCC3, metastasis, EIF4 pathway, downregulation of SMAD2-SMAD3-SMAD4 transcriptional activity, EZH2 targets, ERBB1 receptor proximal pathway, BMI1 targets, signaling by hippo, and oncogenesis by Met (Supplementary Table S6). Some interesting pathways are shown in Figure 7. It’s not containing GSEA analysis for METTL3, IGF2BP1, and IGF2BP3. We did it, but there were no significant results for METTL3, IGF2BP1, and IGF2BP3.

We conducted GSEA analysis in different sample risk score groups based on the LASSO regression model. The GSEA result showed that it is significantly related to cancer survival, oncogenesis by met, gemcitabine resistance, response to UV, HOXC6 targets cancer, recurrent liver cancer, WTAP targets, tumor differentiated well vs. poorly, epithelial mesenchymal transition, hypoxia pathway, TGFB1 targets, cancer meta signature, and so on (Supplementary Table S7). Some interesting pathways are shown in Supplementary Figure S3, and those pathways closely related with tumorigenesis and development.

### The Independent Verification by GEO

The different expression of m6A regulators between cancer tissue and normal tissue, including the m6A methyltransferases, the demethylases, and the m6A-binding proteins were analyzed based on the independent verification by GEO (Supplementary Figure S4 and Supplementary Table S8). In view of some similarities of identified different genes in TCGA data and GEO data, it is believed that the prognostic m6A regulators might not just be due to chance. For example, the overlapping genes that are significant were including RBM15B, KIAA1429, ALKBH5, YTHDF1, IGF2BP 2/3, and HNRNPC. Furthermore, the testing dataset based on GEO showed the different expression of m6A regulators in PC and validate the reliability of the built model based on TCGA. The optimal six-gene signature prognostic model was validated. According to the median risk score, patients from GEO were divided into low- and high-risk score groups (Supplementary Table S9). There was a significant difference in the OS rate between the two groups, and the OS rate of the high-risk score group was significantly lower than that of the low-risk score group (Supplementary Figure S5, *p* = 0.0012).

### Experimental Validation

**FIGURE 8:**
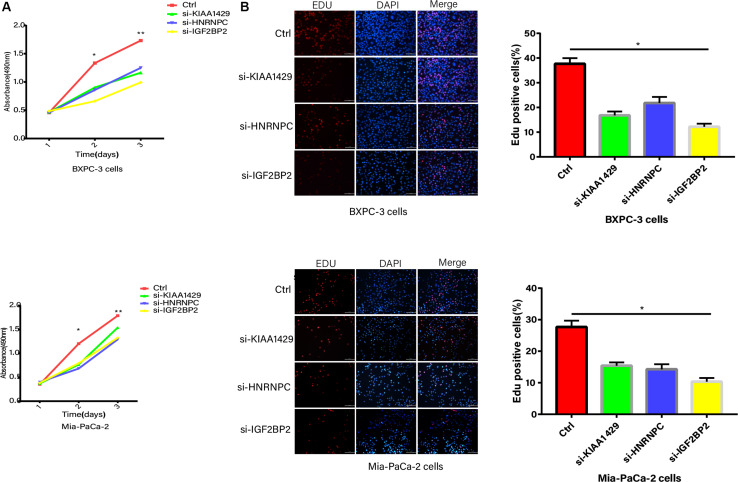
Proliferation abilities validation. **(A)** CCK8 analysis of growth curve in Mia-PaCa-2 and BXPC-3 cells transfected with control and si-RNAs. **(B)** The EdU assay of DNA replication in both Mia-PaCa-2 and BXPC-3 cells transfected with control and si-RNAs. *p < 0.05, **p < 0.01, and ***p < 0.001.

The inhibition of KIAA1429, HNRNPC, and IGF2BP2, respectively, significantly suppressed the proliferation abilities of PC cells based on CCK8 (Figure 8A). The EdU assay further showed that KIAA1429, HNRNPC, and IGF2BP2 inhibitors reduced DNA replication in both Mia-PaCa-2 and BXPC-3 cells (Figure 8B).

## Discussion

Treatment for PC has improved considerably, for example surgery with high success and lower complication rate is better than ever before, novel drug combinations (chemotherapy, target therapy, and immunotherapy) have been shown to improve survival rate, and advances in radiation therapy have achieved less toxicity; however, many researchers are focused on early diagnosis and prompt treatment as PC is still one of the deadliest solid malignancies (Chu et al., 2017). The development of multi-omics has given us a better understanding of the fundamental genetics of PC. These advancements provide hope, but the survival rate of patients with PC is still poor (Cid-Arregui and Juarez, 2015). Biological functions of m^6^A were not studied extensively until around 2012, when major progress was made in the transcriptome profiling of m^6^A through antibody-based immunoprecipitation and high-throughput sequencing (Gan et al., 2019). Moreover, m^6^A regulators were shown to be related with the development of cancer (He et al., 2019). The process of m^6^A modification is reversible through the regulation of m^6^A methyltransferases, demethylases, and binding proteins. A series of m^6^A regulators have been described (Dominissini et al., 2012), including ZC3H13, RBM15/15B, KIAA1429, METTL14, YTHDC1/2, WTAP, METTL3, FTO, ALKBH5, YTHDF1/2/3, HNRNPA2B1, IGF2BP1/2/3, and HNRNPC (Lee et al., 2014). Therefore, it is necessary to explore the influence of m^6^A regulators on PC.

Recent studies have found that the m^6^A modification, when the related enzyme is abnormal, plays various roles in a series of human diseases such as neurological disorders, cancer, and embryonic developmental retardation (Wu et al., 2019). Both coding RNAs and some non-coding RNAs, such as lncRNA, microRNA, tRNA, and rRNA and RNA splice body, were regulated by an m^6^A modification before and after transcription (Yen et al., 2019). N6-methyladenosine modification is closely related to the metabolic processes of RNAs, for example, RNA processing, RNA transfer from the nucleus to the cytoplasm, RNA translation, RNA decay, and the biogenesis of RNA (Liang et al., 2020). The dynamic modification of RNA as a way of regulating genetic information is a new field of study, so there is still a lot of work to be done to understand the underlying mechanisms. Recently, a number of studies have found that m^6^A modifications are associated with cancer, having functions such as helping tumor stem cells to self-renew, promoting the growth and proliferation of cancer cells, and resisting radiotherapy or chemotherapy (Mao et al., 2019). All this evidence indicates that m^6^A regulators may be a target for cancer treatment (Bi et al., 2019; Ianniello et al., 2019). The regulation of m^6^A modifications is a collaboration between methyltransferases, demethylases, and binding proteins. The functions of these proteins in stem cell differentiation, stomach cancer, lung cancer, osteosarcoma, liver cancer, colorectal cancer, leukemia, neuroblastoma, renal cell carcinoma, and breast cancer have been extensivelyreported (Feng et al., 2019; Jin et al., 2019). For example, YTHDF1-deficient mice show an elevated antigen-specific CD8^+^ T cell antitumor response compared with wild-type mice, which indicated that durable neoantigen-specific immunity is regulated by mRNA m^6^A methylation through the m^6^A-binding protein YTHDF1 (Han et al., 2019). It was also reported that some drugs with antitumor activity, such as R-2-hydroxyglutarate (R-2HG), inhibited proliferation/survival of FTO-high cancer cells via targeting FTO/m^6^A/MYC/CEBPA signaling (Su et al., 2018). METTL3 which is independently of METTL14, binds to chromatin, and locates the transcription initiation site of active genes. The promoter bounding METTL3 induces m6A modification in the coding region of the relevant mRNA transcription and enhances its translation by alleviating ribosomal stalling. The gene regulated by METTL3 in this way is necessary for acute myeloid leukemia, suggesting that METTL3 may be a therapeutic target for acute myeloid leukemia (Barbieri et al., 2017). The researchers also found that that m^6^A mRNA demethylation by FTO increases melanoma growth and decreases response to anti-PD-1 blockade immunotherapy (Yang et al., 2019). Knockdown of FTO increased the methylation of m6A in the intrinsic genes of key primary melanoma cells such as PD-1 (PDCD1), CXCR4, SOX10, and so on, leading to increased attenuation of RNA in m6A reader YTHDF2, suggesting that FTO inhibition combined with anti-PD-1 blocking may abate the resistance of melanoma immunotherapy (Yang et al., 2019).

TCGA, a landmark cancer genomics project, described more than 20,000 primary cancers at the molecular level and matched normal samples of 33 cancer types. TCGA generated more than 2.5 petabytes of genome, epigenome, transcriptome and proteome data. The data has already lead to improvements in our ability to diagnose, treat, and prevent cancer (Blum et al., 2018). N6-methyladenosine RNA methylation regulators can lead to malignant progression and impact the prognosis of many kinds of cancer based on the TCGA database. For example, the lasso Cox regression model was applied to identify three m^6^A regulators in bladder cancer. The risk signature was constructed as follows: 0.164FTO - (0.081YTHDC1 + 0.032WTAP), which indicated that the three m^6^A regulators identified might be promising prognostic biomarkers to guide personalized treatment for patients with bladder cancer (Chen et al., 2019). Another study has built up a robust m^6^A regulators-based molecular signature that predicts the prognosis of patients with head and neck squamous cell carcinoma with high accuracy, which might provide important guidance for therapeutic strategies. The results revealed that the expression levels of YTHDF1, METTL3, KIAA1429, YTHDF2, RBM15, METTL14, ALKBH5, FTO, WTAP, and HNRNPC were significantly upregulated in head and neck squamous cell carcinoma samples, while YTHDC2 was remarkably downregulated (Zhao and Cui, 2019). In addition, a study identified two subgroups of gastric cancer (cluster1 and 2) by applying consistency clustering to the m6A regulators. Compared with the cluster1 subgroup, the prognosis of the cluster2 subgroup was poorer, and most of the 13 major m6A regulators were highly expressed in cluster2. This finding provides clues to understand epigenetic modifications of RNA in gastric cancer (Su et al., 2019). However, the prognostic role of m^6^A regulators in PC is poorly understood. In the present study, we are the first to show, by applying consensus clustering to m^6^A regulators, that there are two subgroups of PC (cluster1 and 2). The cluster2 subgroup correlates with a poorer prognosis, which suggests that m^6^A regulators may be promising prognostic biomarkers for PC. Furthermore, the lasso regression analysis identified a six-gene signature prognostic model (KIAA1429, HNRNPC, METTL3, YTHDF1, IGF2BP2, and IGF2BP3). These results agree with the results of previous studies. The major function of IGF2BP2 is to regulate cell metabolism (Huang et al., 2018). However, our results suggest that lncRNA DANCR is a novel target for IGF2BP2 through m^6^A modification in PC, and that it promotes cancer stemness-like properties and PC pathogenesis. Mechanistically, IGF2BP2 serves as a reader for the m^6^A modified DANCR (at adenosine 664), and the definite interaction site provides a novel target for PC therapy (Hu et al., 2019).

We did GSEA for KIAA1429, HNRNPC, and IGF2BP2. Many enrichment pathways were significantly related to cancer pathogenesis. We focused on some important events, for example, pathways of oncogenesis by Met, EIF4 pathway, downregulation of SMAD2-SMAD3-SMAD4 transcriptional activity, EZH2 targets, stemness, well vs. poorly differentiated tumor, epithelial mesenchymal transition, UV response via ERCC3, and metastasis. The identified pathways were consistent with reported data. The importance of m^6^A in the response to ultraviolet DNA damage was demonstrated, and the findings support that m^6^A RNA serves as a beacon for the selective, rapid recruitment of DNA polymerase κ to damage sites to facilitate repair and cell survival (Xiang et al., 2017). Meanwhile, many studies show that m^6^A-related genes work on stemness regulation in tumor relapse. For example, METTL3 was identified as a regulator for terminating murine naïve pluripotency. METTL3 knockout preimplantation epiblasts lead to early embryonic lethality, because it is associated with stability of key naïve pluripotency-promoting transcripts (Geula et al., 2015). Epithelial mesenchymal transition (EMT), as an important cellular program during tumor migration, invasion and metastasis, is also regulated by m^6^A mRNA methylation. N6-methyladenosine-sequencing and functional studies confirm that YTHDF1 mediates m^6^A-increased translation of Snail mRNA (a key transcription factor of EMT) (Lin et al., 2019). Interestingly, the process of m^6^A mRNA methylation was also regulated by cytokines (Li et al., 2017). The TGFβ pathway plays roles in disease through the intracellular effectors SMAD2 and SMAD3. SMAD2/3 promotes binding of the m^6^A methyltransferase complex to a subset of transcripts involved in early cell fate decisions. These aspects of m^6^A methyltransferase signaling could have far-reaching implications in the treatment of many cancers (Bertero et al., 2018).

In conclusion, this study is the first to identify and profile the gene signatures of clinical trait-related m^6^A regulatory genes in PC. We also developed a six-gene signature prognostic model, which might play a crucial role in determining the clinical progression of PC. With the development of m^6^A-sequencing and methylated RNA immunoprecipitation, m^6^A regulatory genes might serve as promising molecular biomarkers for monitoring many kinds of cancers and providing important guidance for selecting therapeutic strategies.

## Data Availability Statement

The datasets generated for this study can be found in The Cancer Genome Atlas (TCGA), https://cancergenome.nih.gov/.

## Author Contributions

JH and LL designed the experiments. ZW and HL performed the experiments, data collection, and analysis. HZ wrote the manuscript. LL revised the manuscript and provided financial support. All authors approved the final version of the manuscript.

## Conflict of Interest

The authors declare that the research was conducted in the absence of any commercial or financial relationships that could be construed as a potential conflict of interest.

## Footnotes

1 https://cancergenome.nih.gov/

2 https://www.ncbi.nlm.nih.gov/geo/

3 http://string-db.org

4 http://www.oncolnc.org/

## Supplementary Material

FIGURE S1 Consensus clustering for pancreatic cancer (PC) tissues. **(A)** Consensus clustering for PC tissues based on the expression of m^6^A regulators (*k* = 3). **(B)** Consensus clustering for PC tissues based on the expression of m^6^A regulators (*k* = 4). **(C)** Consensus clustering cumulative distribution function (CDF) for *k* = 2–4. **(D)** Relative change in area under CDF curve for k = 2–4.

FIGURE S2 **(A,B)** Lasso regression complexity was controlled by lambda using the glmnet R package.

FIGURE S3 Gene set enrichment analysis (GSEA) for high risk score vs. low risk score group. **(A)** GSEA enriched the liver cancer survival pathway. **(B)** GSEA enriched the cancer meta signature. **(C)** GSEA enriched the tumor differentiated well vs. poorly pathway. **(D)** GSEA enriched the epithelial mesenchymal transition pathway. **(E)** GSEA enriched the oncogenesis by MET. **(F)** GSEA enriched the hypoxia pathway.

FIGURE S4 Expression differences of m^6^A RNA methylation regulators in pancreatic cancer based on GEO data. Red and green represent relatively high or low expression, respectively. ^∗^*p* < 0.05, ^∗∗^*p* < 0.01, and ^∗∗∗^*p* < 0.001.

FIGURE S5 Lasso regression validation. **(A)** Lasso regression complexity was controlled by lambda using the glmnet R package. **(B)** Overall survival analysis of the high risk score and low risk score group based on GEO data.

TABLE S1 Gene signatures of m^6^A regulators in pancreatic cancer.

TABLE S2 Sample cluster based on m^6^A regulators in pancreatic cancer.

TABLE S3 PPI network of those m^6^A regulators in pancreatic cancer.

TABLE S4 Lasso regression was constructed examining the relationship between gene signature and pancreatic cancer risk.

TABLE S5 The clinical features of pancreatic cancer and clusters based on consensus clustering method.

TABLE S6 Gene sets enriched in pancreatic cancer by GSEA analysis based on expression of m^6^A regulators (IGF2BP2,KIAA1429, and HNRNPC).

TABLE S7 Gene sets enriched in pancreatic cancer by GSEA analysis in different sample risk groups based on the LASSO regression model.

TABLE S8 Gene signatures of m6A regulators and different expression in pancreatic cancer using GEO.

TABLE S9 Lasso regression was constructed examining the relationship between gene signature and pancreatic cancer risk verified by GEO data.

## Abbreviations

FDR: false discovery rate
GSEA: gene set enrichment analysis
HPDE6-C7: human pancreatic duct epithelial cells
m6A: N6-methyladenosine
OS: overall survival
PC: pancreatic cancer
ROC: receiver operator characteristic curve
TCGA: the cancer genome atlas
YAP1: yes-associated protein 1.

## References

[B1] Alhamzawi R. Ali H. (2018). The Bayesian adaptive lasso regression. *Math. Biosci.* 303 75–82. 10.1016/j.mbs.2018.06.004 29920251

[B2] Ansari D. Gustafsson A. Andersson R. (2015). Update on the management of pancreatic cancer: surgery is not enough. *World J. Gastroenterol.* 21 3157–3165. 10.3748/wjg.v21.i11.3157 25805920 PMC4363743

[B3] Barbieri I. Tzelepis K. Pandolfini L. Shi J. Millan-Zambrano G. Robson S. C. (2017). Promoter-bound METTL3 maintains myeloid leukaemia by m(6)A-dependent translation control. *Nature* 552 126–131. 10.1038/nature24678 29186125 PMC6217924

[B4] Bertero A. Brown S. Madrigal P. Osnato A. Ortmann D. Yiangou L. (2018). The SMAD2/3 interactome reveals that TGFbeta controls m(6)A mRNA methylation in pluripotency. *Nature* 555 256–259. 10.1038/nature25784 29489750 PMC5951268

[B5] Bi Z. Liu Y. Zhao Y. Yao Y. Wu R. Liu Q. (2019). A dynamic reversible RNA N(6) -methyladenosine modification: current status and perspectives. *J. Cell. Physiol.* 234 7948–7956. 10.1002/jcp.28014 30644095

[B6] Blum A. Wang P. Zenklusen J. C. (2018). SnapShot: TCGA-analyzed tumors. *Cell* 173:530 10.1016/j.cell.2018.03.059 29625059

[B7] Chen M. Nie Z. Y. Wen X. H. Gao Y. H. Cao H. Zhang S. F. (2019). m6A RNA methylation regulators can contribute to malignant progression and impact the prognosis of bladder cancer. *Biosci. Rep.* 39:BSR20192892. 10.1042/BSR20192892 PMC6923333 31808521

[B8] Chen X. Xu M. Xu X. Zeng K. Liu X. Sun L. (2020). METTL14 suppresses CRC progression via regulating N6-methyladenosine-dependent primary miR-375 processing. *Mol. Ther.* 28 599–612. 10.1016/j.ymthe.2019.11.016 31839484 PMC7001002

[B9] Chu L. C. Goggins M. G. Fishman E. K. (2017). Diagnosis and detection of pancreatic cancer. *Cancer J.* 23 333–342. 29189329 10.1097/PPO.0000000000000290

[B10] Cid-Arregui A. Juarez V. (2015). Perspectives in the treatment of pancreatic adenocarcinoma. *World J. Gastroenterol.* 21 9297–9316. 26309356 10.3748/wjg.v21.i31.9297 PMC4541382

[B11] Dominissini D. Moshitch-Moshkovitz S. Schwartz S. Salmon-Divon M. Ungar L. Osenberg S. (2012). Topology of the human and mouse m6A RNA methylomes revealed by m6A-seq. *Nature* 485 201–206. 10.1038/nature11112 22575960

[B12] Feng Y. Li Y. Li L. Wang X. Chen Z. (2019). Identification of specific modules and significant genes associated with colon cancer by weighted gene coexpression network analysis. *Mol. Med. Rep.* 20 693–700. 31180534 10.3892/mmr.2019.10295

[B13] Gan H. Hong L. Yang F. Liu D. Jin L. Zheng Q. (2019). [Progress in epigenetic modification of mRNA and the function of m6A modification]. *Sheng Wu Gong Cheng Xue Bao* 35 775–783. 31222996 10.13345/j.cjb.180416

[B14] Geula S. Moshitch-Moshkovitz S. Dominissini D. Mansour A. A. Kol N. Salmon-Divon M. (2015). Stem cells. *m*6A mRNA methylation facilitates resolution of naive pluripotency toward differentiation. *Science* 347 1002–1006. 25569111 10.1126/science.1261417

[B15] Han D. Liu J. Chen C. Dong L. Liu Y. Chang R. (2019). Anti-tumour immunity controlled through mRNA m(6)A methylation and YTHDF1 in dendritic cells. *Nature* 566 270–274. 10.1038/s41586-019-0916-x 30728504 PMC6522227

[B16] He L. Li H. Wu A. Peng Y. Shu G. Yin G. (2019). Functions of N6-methyladenosine and its role in cancer. *Mol. Cancer* 18:176. 10.1186/s12943-019-1109-9 PMC6892141 31801551

[B17] He Y. Hu H. Wang Y. Yuan H. Lu Z. Wu P. (2018). ALKBH5 inhibits pancreatic cancer motility by decreasing long non-coding RNA KCNK15-AS1 methylation. *Cell. Physiol. Biochem.* 48 838–846. 10.1159/000491915 30032148

[B18] Hong K. (2018). Emerging function of N6-methyladenosine in cancer. *Oncol. Lett.* 16 5519–5524. 30344705 10.3892/ol.2018.9395 PMC6176263

[B19] Hu X. Peng W. X. Zhou H. Jiang J. Zhou X. Huang D. (2019). IGF2BP2 regulates DANCR by serving as an N6-methyladenosine reader. *Cell Death Differ.* [Epub ahead of print]. 10.1038/s41418-019-0461-z PMC7244758 31804607

[B20] Huang H. Weng H. Sun W. Qin X. Shi H. Wu H. (2018). Recognition of RNA N(6)-methyladenosine by IGF2BP proteins enhances mRNA stability and translation. *Nat. Cell Biol.* 20 285–295. 10.1038/s41556-018-0045-z 29476152 PMC5826585

[B21] Ianniello Z. Paiardini A. Fatica A. (2019). N(6)-methyladenosine (m(6)A): a promising new molecular target in acute myeloid leukemia. *Front. Oncol.* 9:251 10.3389/fonc.2019.00251 PMC6465620 31024852

[B22] Ilic M. Ilic I. (2016). Epidemiology of pancreatic cancer. *World J. Gastroenterol.* 22 9694–9705. 27956793 10.3748/wjg.v22.i44.9694 PMC5124974

[B23] Jin D. Guo J. Wu Y. Du J. Yang L. Wang X. (2019). m(6)A mRNA methylation initiated by METTL3 directly promotes YAP translation and increases YAP activity by regulating the MALAT1-miR-1914-3p-YAP axis to induce NSCLC drug resistance and metastasis. *J. Hematol. Oncol.* 12:135. 10.1186/s13045-019-0830-6 PMC6902496 31818312

[B24] Koh C. Goh Y. T. Goh W. (2019). Atlas of quantitative single-base-resolution N(6)-methyl-adenine methylomes. *Nat. Commun.* 10:5636. 10.1038/s41467-019-13561-z PMC6904561 31822664

[B25] Lee M. Kim B. Kim V. N. (2014). Emerging roles of RNA modification: m(6)A and U-tail. *Cell* 158 980–987. 10.1016/j.cell.2014.08.005 25171402

[B26] Li H. B. Tong J. Zhu S. Batista P. J. Duffy E. E. Zhao J. (2017). m(6)A mRNA methylation controls T cell homeostasis by targeting the IL-7/STAT5/SOCS pathways. *Nature* 548 338–342. 10.1038/nature23450 28792938 PMC5729908

[B27] Liang Z. Riaz A. Chachar S. Ding Y. Du H. Gu X. (2020). Epigenetic Modifications of mRNA and DNA in plants. *Mol. Plant* 13 14–30. 10.1016/j.molp.2019.12.007 31863849

[B28] Lin X. Chai G. Wu Y. Li J. Chen F. Liu J. (2019). RNA m(6)A methylation regulates the epithelial mesenchymal transition of cancer cells and translation of Snail. *Nat. Commun.* 10:2065. 10.1038/s41467-019-09865-9 PMC6502834 31061416

[B29] Liu Z. X. Li L. M. Sun H. L. Liu S. M. (2018). Link between m6A modification and cancers. *Front. Bioeng. Biotechnol.* 6:89 10.3389/fbioe.2018.00089 PMC6055048 30062093

[B30] Lock E. F. Dunson D. B. (2013). Bayesian consensus clustering. *Bioinformatics* 29 2610–2616. 10.1093/bioinformatics/btt425 23990412 PMC3789539

[B31] Mao Y. Dong L. Liu X. M. Guo J. Ma H. Shen B. (2019). m(6)A in mRNA coding regions promotes translation via the RNA helicase-containing YTHDC2. *Nat. Commun.* 10:5332. 10.1038/s41467-019-13317-9 PMC6877647 31767846

[B32] Mcguigan A. Kelly P. Turkington R. C. Jones C. Coleman H. G. Mccain R. S. (2018). Pancreatic cancer: a review of clinical diagnosis, epidemiology, treatment and outcomes. *World J. Gastroenterol.* 24 4846–4861. 10.3748/wjg.v24.i43.4846 30487695 PMC6250924

[B33] Meyer K. D. Jaffrey S. R. (2017). Rethinking m(6)a readers, writers, and erasers. *Annu. Rev. Cell Dev. Biol.* 33 319–342. 10.1146/annurev-cellbio-100616-060758 28759256 PMC5963928

[B34] Mohammed S. Van Buren G. N. Fisher W. E. (2014). Pancreatic cancer: advances in treatment. *World J. Gastroenterol.* 20 9354–9360. 25071330 10.3748/wjg.v20.i28.9354 PMC4110567

[B35] Niu Y. Lin Z. Wan A. Chen H. Liang H. Sun L. (2019). RNA N6-methyladenosine demethylase FTO promotes breast tumor progression through inhibiting BNIP3. *Mol. Cancer* 18:46. 10.1186/s12943-019-1004-4 PMC6437932 30922314

[B36] Su R. Dong L. Li C. Nachtergaele S. Wunderlich M. Qing Y. (2018). R-2HG exhibits anti-tumor activity by targeting FTO/m(6)A/MYC/CEBPA signaling. *Cell* 172 90–105. 29249359 10.1016/j.cell.2017.11.031 PMC5766423

[B37] Su Y. Huang J. Hu J. (2019). m(6)A RNA methylation regulators contribute to malignant progression and have clinical prognostic impact in gastric cancer. *Front. Oncol.* 9:1038 10.3389/fonc.2019.01038 PMC6813557 31681576

[B38] Taketo K. Konno M. Asai A. Koseki J. Toratani M. Satoh T. (2018). The epitranscriptome m6A writer METTL3 promotes chemo- and radioresistance in pancreatic cancer cells. *Int. J. Oncol.* 52 621–629. 29345285 10.3892/ijo.2017.4219

[B39] Welinsky S. Lucas A. L. (2017). Familial pancreatic cancer and the future of directed screening. *Gut Liver* 11 761–770. 10.5009/gnl16414 28609837 PMC5669591

[B40] Wilkerson M. D. Hayes D. N. (2010). ConsensusClusterPlus: a class discovery tool with confidence assessments and item tracking. *Bioinformatics* 26 1572–1573. 10.1093/bioinformatics/btq170 20427518 PMC2881355

[B41] Wolfgang C. L. Herman J. M. Laheru D. A. Klein A. P. Erdek M. A. Fishman E. K. (2013). Recent progress in pancreatic cancer. *CA Cancer J. Clin.* 63 318–348. 23856911 10.3322/caac.21190 PMC3769458

[B42] Wu F. Cheng W. Zhao F. Tang M. Diao Y. Xu R. (2019). Association of N6-methyladenosine with viruses and related diseases. *Virol. J.* 16:133. 10.1186/s12985-019-1236-3 PMC6849232 31711514

[B43] Xia T. Wu X. Cao M. Zhang P. Shi G. Zhang J. (2019). The RNA m6A methyltransferase METTL3 promotes pancreatic cancer cell proliferation and invasion. *Pathol. Res. Pract.* 215:152666 10.1016/j.prp.2019.152666 31606241

[B44] Xiang Y. Laurent B. Hsu C. H. Nachtergaele S. Lu Z. Sheng W. (2017). RNA m(6)A methylation regulates the ultraviolet-induced DNA damage response. *Nature* 543 573–576. 10.1038/nature21671 28297716 PMC5490984

[B45] Yang S. Wei J. Cui Y. H. Park G. Shah P. Deng Y. (2019). m(6)A mRNA demethylase FTO regulates melanoma tumorigenicity and response to anti-PD-1 blockade. *Nat. Commun.* 10:2782. 10.1038/s41467-019-10669-0 PMC6592937 31239444

[B46] Yen C. J. Yang S. T. Chen R. Y. Huang W. Chayama K. Lee M. H. (2019). Hepatitis B virus X protein (HBx) enhances centrosomal P4.1-associated protein (CPAP) expression to promote hepatocarcinogenesis. *J. Biomed. Sci.* 26:44. 10.1186/s12929-019-0534-9 PMC6551916 31170980

[B47] Yu J. Chen M. Huang H. Zhu J. Song H. Zhu J. (2018). Dynamic m6A modification regulates local translation of mRNA in axons. *Nucleic Acids Res.* 46 1412–1423. 10.1093/nar/gkx1182 29186567 PMC5815124

[B48] Zaccara S. Ries R. J. Jaffrey S. R. (2019). Reading, writing and erasing mRNA methylation. *Nat. Rev. Mol. Cell Biol.* 20 608–624. 10.1038/s41580-019-0168-5 31520073

[B49] Zhao X. Cui L. (2019). Development and validation of a m(6)A RNA methylation regulators-based signature for predicting the prognosis of head and neck squamous cell carcinoma. *Am. J. Cancer Res.* 9 2156–2169. 31720080 PMC6834477

