# Supplementary figures and images for: Gene Signature and Identification of Clinical Trait-Related m6 A Regulators in Pancreatic Cancer
Source: Front Genet. 2020 Jul 10;11:522. doi: 10.3389/fgene.2020.00522 (PMC7367043; doi:10.3389/fgene.2020.00522)

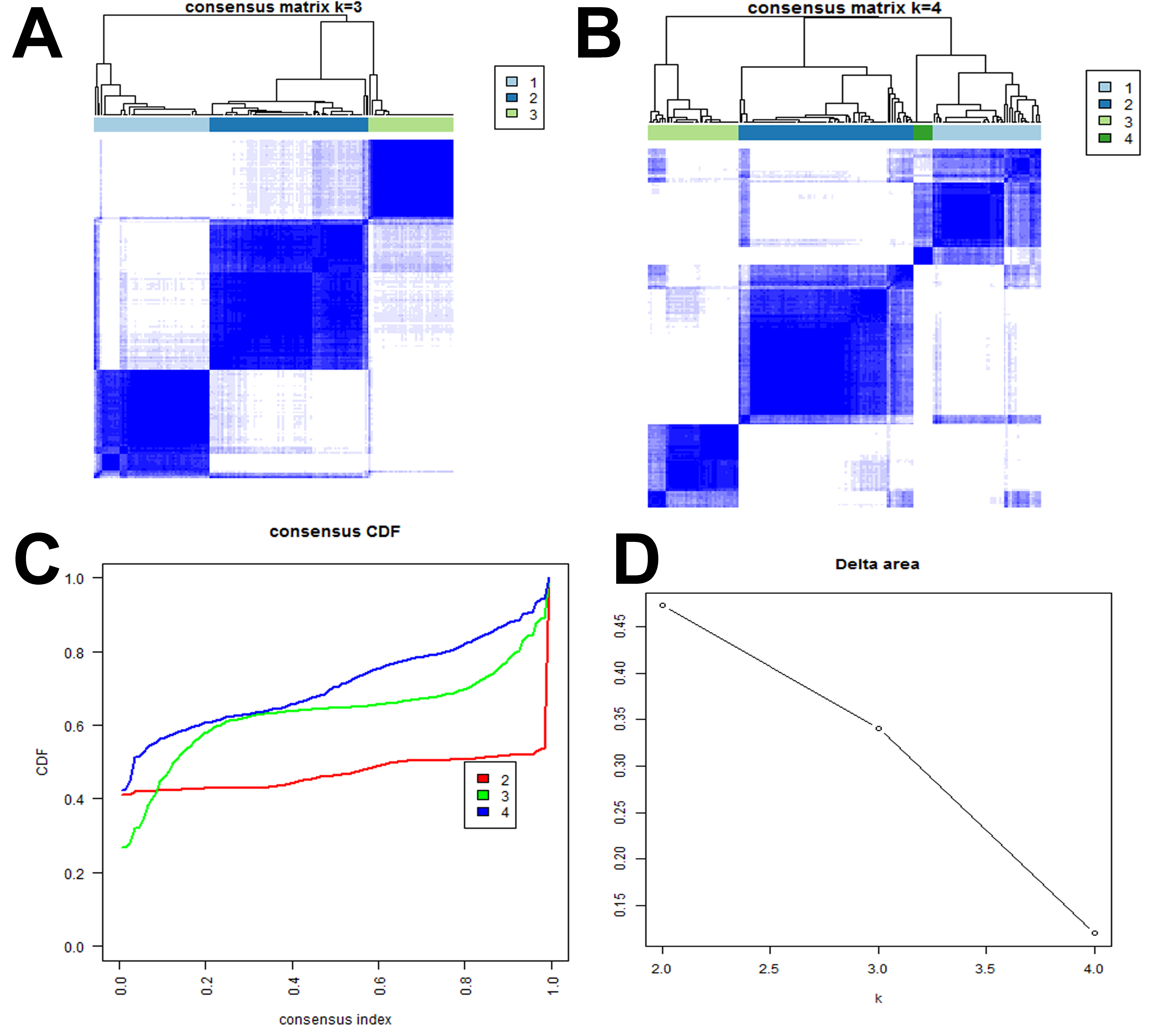

Supplement: FIGURE S1 — Consensus clustering for pancreatic cancer (PC) tissues. (A) Consensus clustering for PC tissues based on the expression of m6A regulators (k = 3). (B) Consensus clustering for PC tissues based on the expression of m6A regulators (k = 4). (C) Consensus clustering cumulative distribution function (CDF) for k = 2–4. (D) Relative change in area under CDF curve for k = 2–4. [file Presentation_1.zip › Supplementary figure 1 (1).tif]

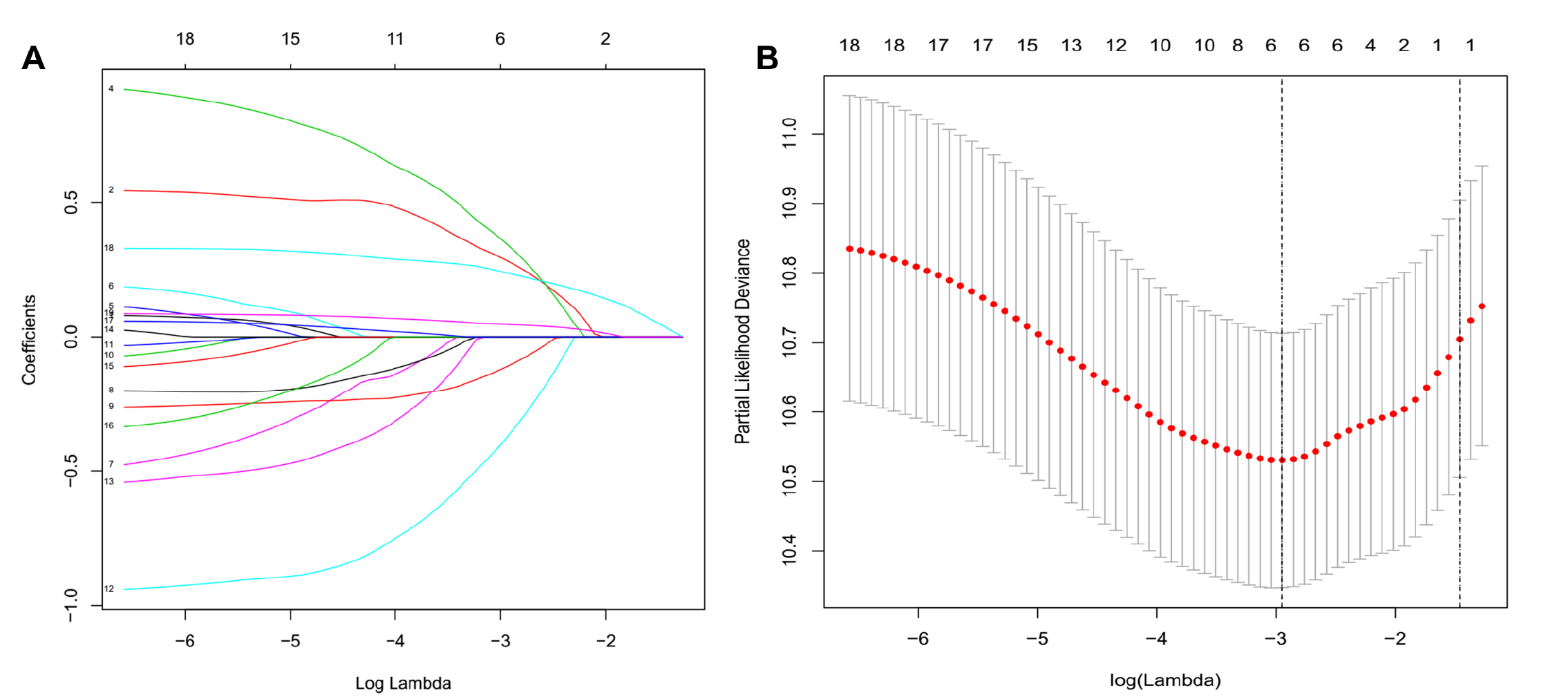

Supplement: FIGURE S1 — Consensus clustering for pancreatic cancer (PC) tissues. (A) Consensus clustering for PC tissues based on the expression of m6A regulators (k = 3). (B) Consensus clustering for PC tissues based on the expression of m6A regulators (k = 4). (C) Consensus clustering cumulative distribution function (CDF) for k = 2–4. (D) Relative change in area under CDF curve for k = 2–4. [file Presentation_1.zip › Supplementary figure 2.tif]

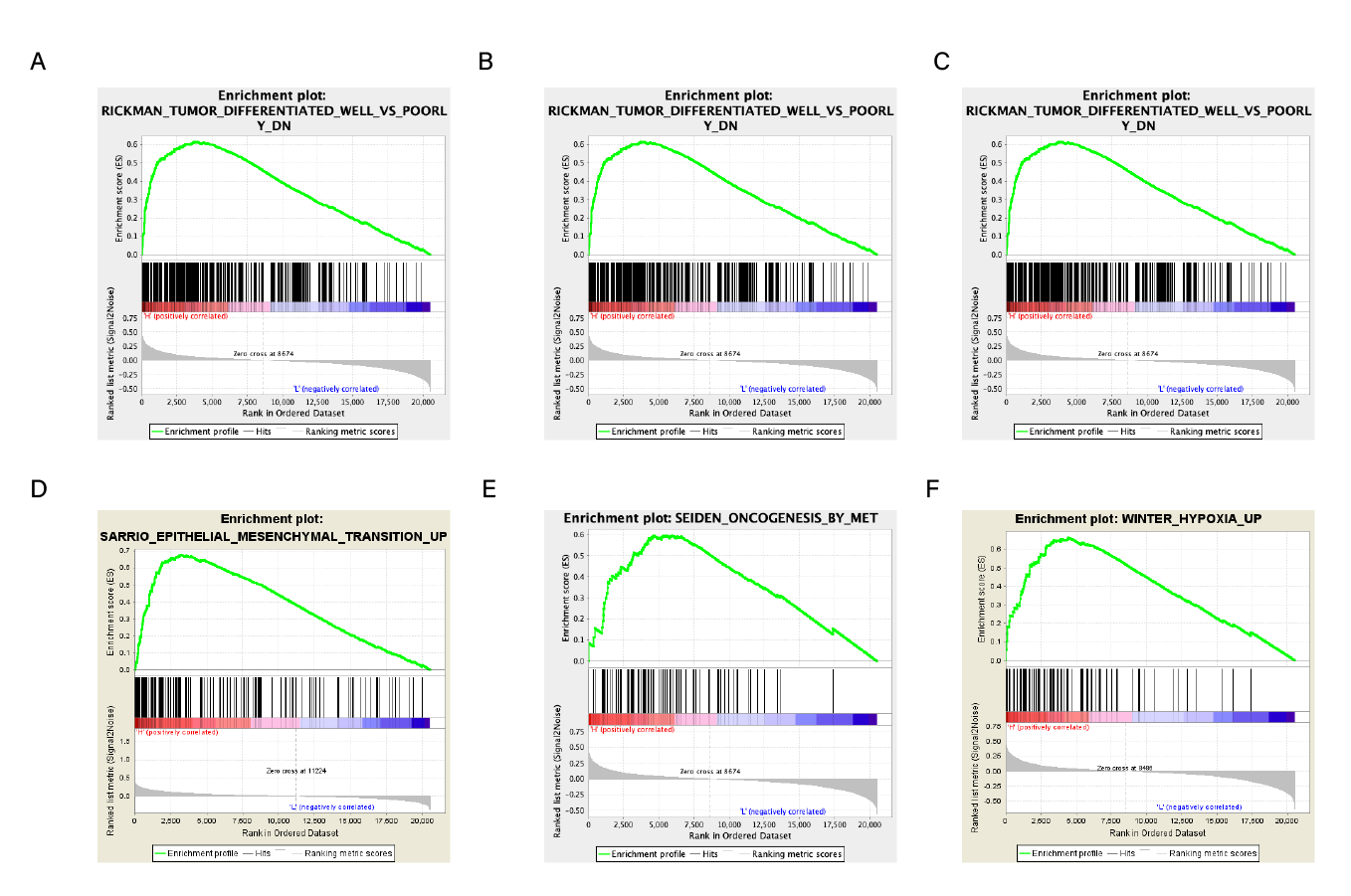

Supplement: FIGURE S1 — Consensus clustering for pancreatic cancer (PC) tissues. (A) Consensus clustering for PC tissues based on the expression of m6A regulators (k = 3). (B) Consensus clustering for PC tissues based on the expression of m6A regulators (k = 4). (C) Consensus clustering cumulative distribution function (CDF) for k = 2–4. (D) Relative change in area under CDF curve for k = 2–4. [file Presentation_1.zip › supplementary figure 3.tif]

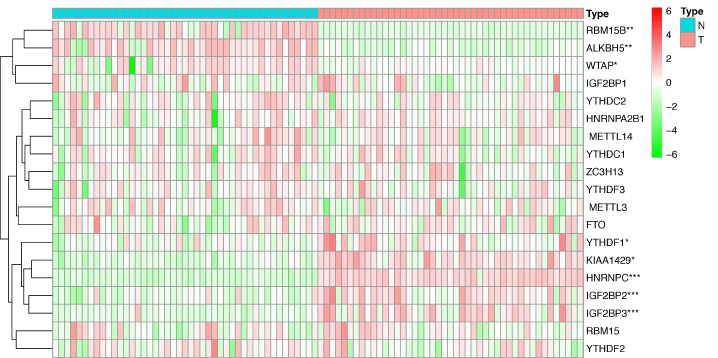

Supplement: FIGURE S1 — Consensus clustering for pancreatic cancer (PC) tissues. (A) Consensus clustering for PC tissues based on the expression of m6A regulators (k = 3). (B) Consensus clustering for PC tissues based on the expression of m6A regulators (k = 4). (C) Consensus clustering cumulative distribution function (CDF) for k = 2–4. (D) Relative change in area under CDF curve for k = 2–4. [file Presentation_1.zip › Supplementary figure 4.tif]

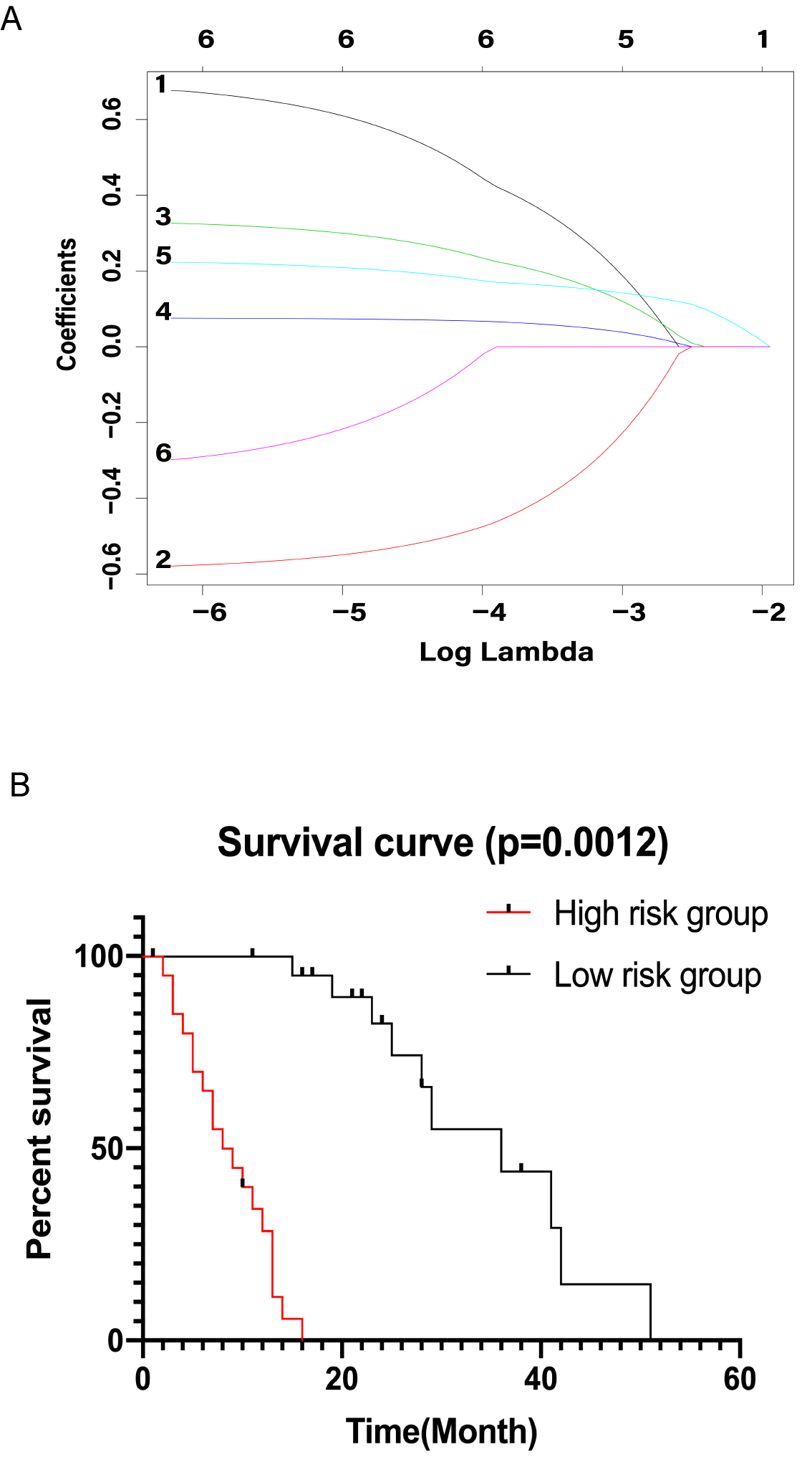

Supplement: FIGURE S1 — Consensus clustering for pancreatic cancer (PC) tissues. (A) Consensus clustering for PC tissues based on the expression of m6A regulators (k = 3). (B) Consensus clustering for PC tissues based on the expression of m6A regulators (k = 4). (C) Consensus clustering cumulative distribution function (CDF) for k = 2–4. (D) Relative change in area under CDF curve for k = 2–4. [file Presentation_1.zip › supplementary figure 5.tif]
